# Effects of dietary fiber sources on bacterial diversity in separate segments of the gastrointestinal tract of native and exotic pig breeds raised in Vietnam

**DOI:** 10.14202/vetworld.2021.2579-2587

**Published:** 2021-10-02

**Authors:** Tran Thi Bich Ngoc, Nguyen Cong Oanh, Tran Thi Thu Hong, Pham Kim Dang

**Affiliations:** 1 Department of Animal Nutrition and Feed, National Institute of Animal Sciences, Hanoi 100000, Vietnam; 2 Excellent Research Team, Faculty of Animal Science, Vietnam National University of Agriculture, Hanoi 131000, Vietnam; 3 Department of Animal Sciences, University of Agriculture and Forestry, Hue University, Hue 530000, Vietnam.

**Keywords:** bacterial diversity, brewer’s grain, cassava by-products, exotic pig, fiber, native pig

## Abstract

**Background and Aim:**

Dietary fiber has distinctive effects on the environment and microbiota of the pig’s intestinal tract. This study was conducted at the naturally ventilated facility of the experimental station, National Institute of Animal Sciences, Vietnam, to examine the effects of fiber sources in diets on the intestinal microbiota of two different pig breeds raised in Vietnam.

**Materials and Methods:**

A total of 18 native and 18 exotic pigs with average initial body weights of 9.5±0.4 and 16.5±0.4 kg, respectively, were each divided into three dietary treatments, including a low-fiber diet containing approximately 200 g NDF per kg dry matter (DM) and two high-fiber diets containing cassava by-products or brewer’s grains containing approximately 300 g NDF per kg DM. At the end of the experiment (28 days), the bacterial diversity of digesta samples collected from the stomach, ileum, and colon segments was analyzed through DGGE analysis of the V3 variable regions of 16S-rDNA and by cloning and sequencing.

**Results:**

Among the diets, significant differences were observed in the number of DNA bands in the stomach between the native and exotic pigs (p<0.05), but not in the ileum and colon. The dietary fiber affected the number of DNA bands in the ileum (p<0.05), but not in the stomach and colon. A significant interaction effect was found between diet and breed on the number of DNA bands in the ileum (p<0.05). Dietary fiber and breed had a greater effect on microbiota in the ileum and colon than that in the stomach.

**Conclusion:**

The fiber sources affected the number of DNA bands in the ileum, and breed affected the number of DNA bands in the stomach. The microbial compositions in the ileum and colon segments were significantly affected by the dietary fiber and breed.

## Introduction

Fiber is a structural component of plants and constitutes an essential element in the diet of animals [1]. Dietary fiber consists of non-starch polysaccharides (NSP) and lignin that cannot be digested in the small intestine by endogenous digestive enzymes. The content of NSP and the soluble/insoluble fractions of NSP in the diets change the bacterial diversity in the large intestine and may be capable of preventing intestinal disorders in pigs [2]. Moreover, fructooligosaccharides are indigestible feed components that affect the gut microbial composition of animals [3] and tend to reduce the amount of *Escherichia coli* and increase the amount of *Bifidobacterium* in the distal colon of pigs [4].

Different fiber sources are known to affect the gut microbial composition of suckling piglets [5], ­rabbits [6], and pregnant sows [7]. In addition, the microbiota compositions of the gut and stomach are significantly affected by the breed and age of animals [8-10]. It has been reported that the breed has a significant effect on the rumen microbial properties [11].

Therefore, we hypothesized that the types of dietary fiber and breeds have important effects on microbiota in the gastrointestinal tract. This study aimed to explore the effects of dietary fiber sources on the bacterial diversity in different segments of the gastrointestinal tract of native and exotic post-weaning pigs.

## Materials and Methods

### Ethical approval

The experiment was approved by the Animal Science Committee of National Institute of Animal Sciences (NIAS), the Ministry of Agricultural and Rural Development, Vietnam.

### Experimental design

The study was carried out at the naturally ventilated facility of the experimental station, NIAS, located in Bac Tu Liem, Hanoi 29909, Vietnam (21°0’18” N latitude and 105°55’57” E longitude). A total of 36 animals, including 18 native Mong Cai and 18 exotic (Landrace × Yorkshire) pigs, aged approximately 60 days, were used in this experiment. The initial body weights (IBWs) of the native and exotic pigs were 9.5±0.4 and 16.5±0.4 kg, respectively. A completely randomized 2×3 factorial design was used with the two breeds (native and exotic) and three diets (a low-fiber diet [LFD] and two high-fiber diets consisting of either cassava by-products [HFC] or brewer’s grain [HFB]). For each breed, the animals were evenly divided into three dietary treatments according to the IBW and sex by treatment. Each treatment had six replicate pens with one pig per pen. Animals were housed individually in concrete-floored pens (1.8 m×0.8 m), fully covered with wooden slats. All pigs were vaccinated against classical swine fever, pasteurellosis, pneumonia, and paratyphoid before starting the experiment.

The experimental diets were formulated on the basis of the primary raw materials, including maize, soybean meal, rice bran, fish meal, and cassava by-products (CB) or brewer’s grain (BG) (Table-1). The LFD diet was incorporated without CB and BG and contained approximately 200 g NDF/kg dry matter (DM), whereas CB and BG were incorporated into the HFC and HFB diets to obtain approximately 300 g NDF/kg DM (Table-2) [12]. The diets were incorporated to meet or exceed the nutrient requirements for post-weaning pigs [13]. The duration of the experiment was 28 days.

**Table-1:** Ingredient composition (%DM) of the experimental diets.

Ingredients	Treatment^a^		

	LFD	HFC	HFB
Maize	51.6	11.6	36.5
Soybean meal	23.0	27.0	9.20
Fish meal	5.0	6.0	2.0
Rice bran	17.0	15.0	15.0
Cassava by-products	-	35.0	-
Brewer’s grain	-	-	30.0
Soybean oil	-	2.0	4.0
Limestone	1.5	1.5	1.5
Dicalcium phosphate	1.0	1.0	1.0
Mineral-vitamin premix	0.3	0.3	0.3
DL-methionine	0.3	0.3	0.2
NaCl	0.3	0.3	0.3
Total	100	100	100

a LFD=Low fiber diet, HFC: High fiber diet containing cassava by-products, HFB=High fiber diet containing brewer’s grain

**Table-2:** Analyzed chemical composition (% DM) of the experimental diets.

Items	Treatment		

	LFD	HFC	HFB
Dry matter	89.3	90.6	91.6
Crude protein	20.9	20.0	20.2
Lysine	1.21	1.20	1.21
Methionine+Cystine	0.99	0.89	0.93
Threonine	0.82	0.78	0.82
Tryptophan	0.28	0.25	0.32
NDF	22.2	29.2	30.1
Cellulose	7.39	9.82	10.5
T-NSP	20.5	24.4	26.4
I-NSP	16.0	19.2	23.3
S-NSP	4.48	5.19	3.17
Klason lignin	4.93	4.30	6.77
DF	25.4	28.7	33.2
Starch	35.5	32.0	26.2
GE (MJ/kg DM)	17.4	17.3	17.6
ME (MJ/kg DM)^b^	13.8	12.9	12.6

I-NSP=Insoluble non-starch polysaccharides, S-NSP=Soluble non-starch polysaccharides, T-NSP=Total non-starch polysaccharides, DF=Dietary fiber, GE=Gross energy, ME=Metabolizable energy. ^a^LFD=Low fiber diet, HFC=High fiber diet containing cassava by-products, HFB=High fiber diet containing brewer’s grain.

b Calculated based on chemical composition and nutrient values of animal feeds in Vietnam [12]

### Collection of digesta samples

All experimental pigs were slaughtered 4 h after morning feeding and thiopental injection. Individual digesta samples from the stomach, ileum, and colon sections were collected immediately into sterile tubes, placed on ice in an insulation box, and transferred to the laboratory. The digesta samples were frozen and stored at −20°C until further DNA extraction.

#### Determination of general microbial diversity

DNA isolation

DNA isolation of the stomach, ileum, and colon samples was conducted using the AccuPrep^®^ Stool DNA Extraction Kit (Bioneer, Korea) according to the manufacturer’s instructions. Total RNA was treated with the enzyme ribonuclease. The concentration and purity of DNA were checked using the NanoDrop^®^ ND-1000 spectrophotometer (Wilmington, USA). After isolation, the DNA samples were stored at −20°C until polymerase chain reaction (PCR) amplification.

PCR amplification

PCR amplification of the V3 hypervariable region of the bacterial *16S rDNA* gene was conducted using the forward primer 341F with GC clamp at the 5' end (5'-CGC CCGCCGCGCGCGGCGGGCGGGGCGGGG GCACGGGGGGCCTACGGGA GGCAGCAG-3') and the reverse primer 518R (5'-ATTACCGCGGCTGCTGG-3'). The primers 341F and 518R were designed on the basis of the position of the nucleotides 341-518 of *16S rDNA* in the *E. coli* gene [14,15]. Approximately 35 ng of DNA template was used for PCR. DNA was amplified as described by Hong *et al*. [16]. PCR products were tested by 2% agarose gel electrophoresis. Gels were stained with 0.5 μg/mL ethidium bromide, checked under ultraviolet (UV) light, and photographed under the Gel DOC™ 2000 Gel Documentation System (Bio-Rad, USA).

DGGE

The amplified PCR products were separated using the Bio-Rad Dcode™ Universal Mutation Detection System (Bio-Rad, USA), with the size of the gel being 16 cm× 20 cm. The gels consisted of 10% polyacrylamide and 30-60% linear gradients of the denaturant. The concentration of ammonium persulfate solution was 0.1% (w/v), and the concentration of tetramethylethylenediamine was 0.1% (v/v). Furthermore, 2% (v/v) glycerol was used to increase the durability of gels. A total of 20 μL of the PCR product was mixed with the loading dye and added to a well. Electrophoresis was conducted at 60°C for 17 h at 85 V, and the DGGE gels were then stained with 0.5 μg/mL ethidium bromide for 30 min, checked under UV light, and photographed under the Gel DOC™ 2000 Gel Documentation System (Bio-Rad, USA). Selected DGGE bands were cut out from a gel using a sterile scalpel, placed into tubes containing 30 mL distilled water, and stored overnight at 4°C. Next, pure DNA from these tubes was collected, transferred to new tubes, and stored at −20°C for further analysis.

Gel analysis

DGGE images were analyzed using Quantity One 4.5.0 (Bio-Rad, USA). Ladder 1 kb marker was used as the reference system. The number of bands in each DGGE profile was determined as an indicator of richness, and comparisons between the dietary treatments were conducted. The Dice coefficient was used for comparing the fingerprint profiles of DGGE images using NTSYSpc version 2.1 (NTSYS-PC 2.10, Applied Biostatistics, Setauket, NY, USA).

Amplification of DNA bands from DGGE gels

A total of 1 μL of the eluate were used as a template in a PCR with the primers 341F without GC clamp (5'-CCTACGGGAGGCAGCAG-3') and 518R. The PCR was conducted in a Bio-Rad thermos cycler (MJ Mini Personal Thermal Cycler, USA) using the conditions as described by Hong *et al*. [16]. The PCR products were stored at 4°C for cloning.

Cloning

The amplified molecules were cloned into the vector pTZ57R/T using the InsTAclone™ PCR cloning Kit (Fermentas, Canada). Thereafter, the plasmids were transformed into *E. coli* DH5a. X-Gal and IPTG were used for blue/white colony selection on LB plates containing 50 μg/mL ampicillin.

Analyzing positive colonies

White colonies for each cloned band were randomly selected from the transformation plates. Cells from each colony were transferred using a sterile toothpick into a PCR tube. The PCR was conducted with the specific primers M13F (5'-GTAAAACGACGGCCAG-3') and M13R (5'-CAGGAAACAGCTATGAC-3'). The program for amplification was exactly similar to the amplification with the primers 341F/518R without GC clamp as described earlier, but the final extension at 72°C was conducted for 7 min. The PCR products were checked by electrophoresis in 2% agarose gel and purified using the AccuPrep PCR Purification Kit (Bioneer, Korea).

Sequencing

Sequencing was conducted by Molecular Cloning Laboratories using the purified PCR product as the template and the primer M13F or M13R as described by Hong *et al*. [16].

DNA sequence analysis

Results obtained from the sequencing of DNA samples were compared with sequences available in the NCBI using the BLASTn tool [17].

### Statistical analysis

Experimental data were analyzed using the MIXED procedure of the SAS software (Version 9.0, Institute Inc., Cary, NC, USA). An individual pig was considered as an experimental unit. Repeated measures conducted on the same experimental unit as a similar model were used but included the effect of a compound symmetry structure of covariance. Group means that showed statistical differences at the probability level of p<0.05 were compared using Tukey’s multiple comparison procedures. In addition, the Dice similarity coefficient was calculated from the DGGE profiles of bacterial *16S rDNA* PCR amplification products. The level of similarity between each pair of groups was named in their corresponding row and column. The Dice coefficient did not relate to the p-value, which is associated with statistically significant differences. Instead, the DGGE profile of each group comprised DNA bands that appeared on bands belonging to that group.

## Results

### Bacterial diversity at separate segments in the gastrointestinal tract

The PCR products obtained from genomic bacterial DNA isolated from the segments of the stomach, ileum, and colon as the template were clear and without by-products. The final PCR product length was approximately 200 bp, as determined by 2% agarose gel electrophoresis. DGGE profiles were produced from samples obtained from the stomach, ileum, and colon of pigs fed with different experimental diets. One remarkable feature of the pig microbiota was relatively different among the individuals. As shown in Table-3, there was a significant difference in the number of DNA bands in the stomach between native and exotic pigs (p<0.05), with a higher value for exotic pigs, but not in the ileum and colon. The number of DNA bands in the ileum was significantly different among diets (p<0.05), with the highest value with the HFB diet, medium value with the HFC diet, and the lowest value with the LFD diet. An interaction effect was observed between diet and breed on the number of DNA bands in the ileum segment (p<0.05), such that diet had an effect on the number of DNA bands in exotic pigs, but not in native pigs. The number of DNA bands in the stomach samples was twice that in the colon and ileum samples within breed.

**Table-3:** Number of DNA bands on DGGE gels in stomach, ileum, colon segments of native, and exotic pigs.

Item	Stomach	Ileum	Colon
Breed			
Exotic breed	15.91^a^	6.92	5.42
Native breed	11.17^b^	6.17	6.67
Diet^a^			
LFD			
Exotic breed	13.75	3.25^b^	4.00
Native breed	10.75	4.75^b^	5.25
HFC			
Exotic breed	19.50	6.50^ab^	5.00
Native breed	11.50	8.25^a^	7.25
HFB			
Exotic breed	14.50	11.00^a^	7.25
Native breed	11.25	5.50^b^	7.50
SEM	2.71	1.40	1.73
p-value			
Breed	*	NS	NS
Diet	NS	*	NS
Breed*Diet	NS	*	NS

* p<0.05, Different superscript letters are significantly different (p<0.05).

NS = Non-significant.

a >LFD = Low fiber diet, HFC = High fiber diet containing cassava by-products, HFB = High fiber diet containing brewer’s grain

### Gene diversity at different segments in the gastrointestinal tract

As shown in Table-4, the intestinal microbial diversity in native and exotic pigs fed with different diets was extremely different. However, the coefficient of gene diversity within breed was higher in the stomach site and lower in the ileum and colon sites. Fiber source and level had a greater impact on microbiota in the ileum and colon samples than that in the stomach samples. Within breed, the coefficient of gene diversity in the gastrointestinal tract was higher in each breed than in combination with two breeds.

**Table-4:** Dice coefficient in gastrointestinal tract of native and exotic pigs fed diets containing different fiber source and level.

Breed	Diet^a^	Native breed			Exotic breed		
	
		LFD	HFC	HFB	LFD	HFC	HFB

Stomach site							
Native breed	LFD	1.00					
	HFC	0.85	1.00				
	HFB	0.71	0.81	1.00			
Exotic breed	LFD	0.30	0.37	0.36	1.00		
	HFC	0.33	0.40	0.39	0.85	1.00	
	HFB	0.34	0.41	0.44	0.86	0.87	1.00

**Ileum site**							

Native breed	LFD	1.00					
	HFC	0.48	1.00				
	HFB	0.63	0.62	1.00			
Exotic breed	LFD	0.10	0.30	0.20	1.00		
	HFC	0.15	0.36	0.23	0.50	1.00	
	HFB	0.19	0.42	0.19	0.55	0.69	1.00

**Colon site**							

Native breed	LFD	1.00					
	HFC	0.55	1.00				
	HFB	0.64	0.50	1.00			
Exotic breed	LFD	0.31	0.29	0.38	1.00		
	HFC	0.36	0.38	0.55	0.47	1.00	
	HFB	0.41	0.47	0.50	0.42	0.59	1.00

a LFD=Low fiber diet, HFC=High fiber diet containing cassava by-products, HFB=High fiber diet containing brewer’s grain

### Identification of cloned 16S rDNA sequence in DGGE profiles

To obtain more detailed information about microbiota at different segments in the gastrointestinal tract of the two pig breeds fed with diets with different fiber sources and levels, the DNA bands were cut from DGGE gels, cloned, and sequenced. A total of 120 predominant DNA bands from six gels (20 dominant DNA bands from each gel) were examined and reamplified with the primers 341F (without GC clamp) and 518R. Then, the PCR products were cloned into the vector pTZ57R/T and transformed into *E. coli* DH5a. The result showed that all DNA bands cut from DGGE gels were cloned. The results of the sequence of DNA bands from different gastrointestinal sites of experimental post-weaning pigs revealed that most of the sequences of DNA bands matched with the V3 variable region of *16S rDNA* of bacteria in the GenBank database.

In the stomach of native pigs, among 20 sequences matching the dominant bands (Table-5A), seven demonstrated high similarity (99-100%) with a known sequence of *Lactobacillus* in GenBank; six demonstrated 100% similarity with a known sequence of *Megasphaera elsdenii*; four showed 97% and 96% similarity with the known sequences of *Prevotella ruminicola* and *Prevotella* spp., respectively; two demonstrated 97% similarity with a known sequence of *Selenomonas* spp.; and one clone did not match any known sequence in GenBank. For exotic pigs, among the 20 sequences matching the dominant bands (Table-6A), one sequence demonstrated high similarity with a known sequence of *Bifidobacterium* in GenBank; six sequences showed 99-100% similarity with a sequence of *Lactobacillus*; one sequence displayed 97% similarity with a sequence of *Helcococcus ovis*; two sequences showed 98% similarity with a sequence of *M. elsdenii*; three sequences demonstrated 98% similarity with a sequence of *Selenomonas* spp.; three sequences showed 96%, 98%, and 99% similarity with the sequences of *P. ruminicola*, *Mitsuokella jalaludinii*, and *Syntrophococcus* spp., respectively; and four other clones did not match any known sequence in GenBank.

**Table-5A:** *16S rRNA* gene sequences of the strong DNA bands from stomach of native pigs detected by DGGE and cloning technique.

S. No.	Code of DNA bands	Species	ID number	Identity (%)	E-value	DNA length (bp)	Diet		

							LFD	HFC	HFB
1	d17	*Lactobacillus delbrueckii subsp. bulgaricus*	CP000156	98	8e-93	194	X	X	X
2	d1, d19	*Lactobacillus spp. Lactobacillus equicursoris*	FR681900 AB290830	99	2e-94	194	O	O	X
3	d3, d10, d15	*Lactobacillus helveticus Lactobacillus gallinarum Lactobacillus crispatus Lactobacillus amylovorus Lactobacillus acidophilus*	JF728275 AB596947 HQ718591 CP002338 CP000156	100	4e-96	194	X	X	X
4	d6	*Lactobacillus mucosae*	FR693800	100	4e-96	194	X	X	X
5	d2, d5, d7, d8, d9, d18	*Megasphaera elsdenii*	EU728750	100	2e-93	194	X	X	X
6	d12, d16	*Prevotella ruminicola*	AF218618	97	4e-86	189	X	X	X
7	d11, d13	*Prevotella spp.*	EU728713	96	2e-83	189	X	X	X
8	d14, d20	*Selenomonas spp.*	CP002559	97	5e-90	193	X	X	X

X: Detected, O: Non-detected. LFD=Low fiber diet, HFC=High fiber diet containing cassava by-products, HFB=High fiber diet containing brewer’s grain

**Table-5B:** *16S rRNA* gene sequences of the strong DNA bands from ileum of native pigs detected by DGGE and cloning technique.

S. No.	Code of DNA bands	Species	ID number	Identity (%)	E-value	DNA length (bp)	Diet		

							LFD	HFC	HFB
1	h51	*Bifidobacterium aerophilum*	AY174107	100	2e-68	146	X	X	X
2	h58	*Bifidobacterium spp.*	HQ842703	100	1e-85	174	X	X	X
3	h76’	*Bifidobacterium spp. Bifidobacterium thermophilum Bifidobacterium boum Bifidobacterium ruminantium*	JF519688 GU361834 GU361814 GQ438820	100	3e-87	178	X	X	X
4	h52, h59	*Clostridium cadaveris Clostridium spp. Clostridium disporicum Clostridium quinii*	AB542932 JF269096 DQ855943 NR026149	100	2e-82	169	X	X	X
5	h79, h80, h54, h77	*Clostridium sordellii Clostridium bifermentans Clostridium spp. Clostridium difficile*	HQ259293 AB618787 AB596881 HQ328072	100	2e-82	169	X	X	X
6	h63’	*Cupriavidus spp.*	HQ417092	100	4e-96	194	X	X	O
7	h57	*Gemella haemolysans*	HM103931	98	4e-91	194	O	O	X
8	h69, h70, h71, h71’	*Lactobacillus helveticus Lactobacillus gallinarum Lactobacillus crispatus Lactobacillus amylovorus Lactobacillus acidophilus*	JF728275 AB596947 HQ718591 CP002338 CP000156	100	4e-96	194	X	X	X
9	h72	*Megasphaera elsdenii*	EU728750	100	2e-93	194	X	X	X
10	h68	*Mycoplasma sualvi*	AF412988	100	4e-96	194	O	X	O

X: Detected, O: Non-detected. LFD=Low fiber diet, HFC=High fiber diet containing cassava by-products, HFB=High fiber diet containing brewer’s grain

**Table-5C:** *16S rRNA* gene sequences of the strong DNA bands from colon of native pigs detected by DGGE and cloning technique.

S. No.	Code of DNA bands	Species	ID number	Identity (%)	E-value	DNA length (bp)	Diet		

							LFD	HFC	HFB
1	k131	*Butyrivibrio fibrisolvens*	AM039822	99	3e-81	170	O	O	X
2	k146, k147	*Clostridium sordellii Clostridium bifermentans Clostridium spp. Clostridium difficile*	HQ259293 AB618787 AB596881 HQ328072	100	9e-82	169	O	X	O
3	k136	*Coprococcus spp. Coprococcus eutactus*	EU728700 EF031543	98	5e-79	169	O	O	X
4	k157, k149	*Dialister succinatiphilus*	AB370249	97	1e-86	195	X	X	O
5	k138	*Escherichia coli strain ATCC Shigella sonnei Shigella flexner*	JF508268 HQ407271 HQ407267	100	4e-96	194	O	X	X
6	k141, k156	*Lactobacillus helveticus Lactobacillus gallinarum Lactobacillus crispatus Lactobacillus amylovorus Lactobacillus acidophilus*	JF728275 AB596947 HQ718591 CP002338 CP000156	100	4e-96	194	X	X	X
7	k150	*Mitsuokella jalaludinii Mitsuokella multacida*	NR028840 NR027596	96	1e-86	195	O	X	O
8	k134	*Ruminococcus spp.*	FJ889653	97	5e-74	169	X	X	X
9	k155	*Eubacterium rectale*	AB626630	99	2e-62	136	X	X	X

X: Detected, O: Non-detected. LFD=Low fiber diet, HFC=High fiber diet containing cassava by-products, HFB=High fiber diet containing brewer’s grain

**Table-6A:** *16S rRNA* gene sequences of the strong DNA bands from stomach of exotic pigs detected by DGGE and cloning technique.

S. No.	Code of DNA bands	Species	ID number	Identity (%)	E value	DNA length (bp)	Diet		

							LFD	HFC	HFB
1	d232	*Bifidobacterium thermophilum Bifidobacterium boum ifidobacterium ruminantium*	GU361834 GU361814 GQ438820	100	1e 79	178	X	X	X
2	d222	*Lactobacillus reuteri Lactobacillus vaginalis*	JF268325 AB596995	100	4e 96	194	X	X	X
3	d275	*Helcococcus ovis*	AB542080	97	1e 74	167	X	X	X
4	d223, d228, d230, d259	*Lactobacillus helveticus Lactobacillus gallinarum Lactobacillus crispatus Lactobacillus amylovorus Lactobacillus acidophilus*	JF728275 AB596947 HQ718591 CP002338 CP002559	100	1e 95	194	X	X	X
5	d220	*Lactobacillus equicursoris*	FR681900	99	2e 94	194	X	O	O
6	d270, d287	*Megasphaera elsdenii*	AB298908	98	1e 96	195	X	X	X
7	d227	*Mitsuokella jalaludinii Mitsuokella multacida*	NR_028840 NR_027596	98	2e 93	195	X	X	X
8	d224	*Prevotella ruminicola*	AF218618	96	2e 83	199	O	X	X
9	d260, d247, d267	*Selenomonas spp.*	GQ332234	98	5e 75	195	X	X	X
10	d248	*Syntrophococcus spp.*	GU045475	99	1e 80	169	X	X	X

X: Detected, O: Non detected. LFD = Low fiber diet, HFC = High fiber diet containing cassava by products, HFB = High fiber diet containing brewer’s grain

**Table-6B:** *16S rRNA* gene sequences of the strong DNA bands from ileum of exotic pigs detected by DGGE and cloning technique.

S. No.	Code of DNA bands	Species	ID number	Identity (%)	E-value	DNA length (bp)	Diet		

							LFD	HFC	HFB
1	h116	*Bifidobacterium aerophilum*	AY174107	99	2e-83	174	X	X	X
2	h123	*Clostridium quinii Clostridium aurantibutyricum Clostridium aurantibutyricum*	AB542932 NR026149 X68183	100	2e-82	169	O	O	X
3	h117, h124	*Clostridium sordellii Clostridium bifermentans*	HQ259293 AB601079	100	2e-82	169	X	X	X
4	h105	*Lactococcus garvieae*	HQ721279	99	5e-95	195	O	X	X
5	h99	*Lactococcus lactis*	FJ429979	100	4e-94	194	O	X	X
6	h101	*Streptococcus equinus Streptococcus macedonicus Streptococcus pasteurianus Streptococcus macedonicus*	GU222444 HQ721250 AP012054 AB563258	100	1e-96	195	X	X	X
7	h100	*Streptococcus hyointestinalis*	EU728763	100	1e-96	195	O	X	O
8	h94, h95	*Veillonella magna*	EU096495	98	2e-93	195	O	X	X

X: Detected, O: Non-detected. LFD=Low fiber diet, HFC=High fiber diet containing cassava by-products, HFB=High fiber diet containing brewer’s grain

**Table-6C:** *16S rRNA* gene sequences of the strong DNA bands from colon of exotic pigs detected by DGGE and cloning technique.

S. No.	Code of DNA bands	Species	ID number	Identity (%)	E-value	DNA length (bp)	Diet		

							LFD	HFC	HFB
1	k175	*Butyrivibrio fibrisolvens Roseburia intestinalis Lachnobacterium*	AM039822 HM007565 AJ518873	98	2e-93	171	X	X	X
2	k183	*Escherichia coli Shigella sonnei Shigella flexner*	JF508268 HQ407271 HQ407267	100	1e-95	194	O	O	X
3	k198	*Eubacterium coprostanoligenes*	HM037995	98	4e-96	170	O	X	O
4	k201, k214	*Lactobacillus helveticus Lactobacillus gallinarum Lactobacillus crispatus Lactobacillus amylovorus*	JF728275 AB596947 HQ718591 CP002338	100	1e-93	197	X	X	X
5	k184	*Lactobacillus johnsonii*	CP002464	100	1e-96	194	O	O	X
6	k213	*Megasphaera elsdenii*	AB298908	98	2e-82	195	O	X	X
7	k189, k185	*Streptococcus equinus Streptococcus macedonicus*	GU222444 HQ721250	100	1e-94	195	O	X	X

X: Detected, O: Non-detected. LFD=Low fibre diet, HFC=High fibre diet containing cassava by-products, HFB=High fiber diet containing brewer’s grain

In the ileum of native pigs, most sequences matched with *Clostridium* (six sequences), followed by *Lactobacillus* (four sequences) and *Bifidobacterium* (three sequences) in GenBank, with a similarity of 100% (Table-5B). For exotic pigs, 10 of 20 sequences matched with known sequences in GenBank, whereas the remaining ten sequences belonged to uncultured bacteria. Six sequences matched with a full similarity of 100%, and four sequences demonstrated 98-99% similarity (Table-6B). The ileum samples showed a group of beneficial bacteria such as *Veillonella magna*, *Bifidobacterium*, and *Lactococcus lactis*.

In the colon samples of native pigs, 20 sequences matched with the V3 variable region of *16S rDNA* of bacteria in GenBank, in which 12 sequences matched with known bacteria were divided into ten different bacterial groups, and the remaining eight sequences matched with uncultured and unknown bacteria (Table-5C). The sequences that matched known bacteria belonged to *Clostridium* (two sequences), *Lactobacillus* (two sequences), and *Dialister* (two sequences). This result was similar to that observed in the ileum; the dominant bands in the DGGE gel matched with the sequence of *Lactobacillus*, and other bands were not clear. *Lactobacillus*, *Ruminococcus*, and *Eubacterium* were found in the pig colon, and *Lactobacillus* was the most popular bacterium in the colon of native pigs. *Butyrivibrio*, *Coprococcus*, *Clostridium*, and *Mitsuokella* were also found in an individual, which were not considered to be specific bacteria in the colon of native pigs. For exotic pigs, nine of 20 sequences matched with *16S rDNA* of bacteria in GenBank, and the remaining 11 sequences matched with uncultured bacteria. Most sequences matched with high similarity between 98% and 100% compared with sequences in GenBank (Table-6C). Three sequences were recognized as belonging to some species (*L. johnsonii*, *Eubacterium coprostanoligenes*, and *M. elsdenii*), and six sequences could not be distinguished between species (*Streptococcus* spp., *Lactobacillus* spp., and *Shigella* spp.).

## Discussion

This study showed that *Lactobacillus*, *Megasphaera*, and *Prevotella* were present in the stomach of native and exotic pigs fed with the three experimental diets. Supporting this finding, the previous studies have reported that these bacterial species were dominant in the digestive tract of pigs [16,18,19]. The DNA bands d8 and d9 in 12 digesta samples collected from the stomach of native pigs matched with the sequence of *M. elsdenii*. Similarly, *M. elsdenii* (DNA bands 270 and 287) was found in all samples collected from the stomach of exotic pigs fed with the three diets. *Selenomonas* spp. also was detected in both native and exotic pigs fed with the three diets. A previous study [20] reported that *Lactobacillus* and *M. elsdenii* were dominant in the stomach of pigs fed with a coarse-non pelleted diet, consistent with our study. Interestingly, *Lactobacillus* and *Bifidobacterium* were detected in the stomach samples of exotic pigs, whereas only *Lactobacillus* was detected in native pigs. This result can be attributed to the environmental factors in the stomach between the native pig breed and exotic pig breed, causing a difference in microbiota composition. *Lactobacillus* and *Bifidobacterium* are known to have important roles in the digestive tract of pigs as they can prevent contamination with harmful bacteria, reduce diarrhea, and regulate the immunological system [21]. The development of these bacteria creates a better environment for the growth of beneficial bacteria and the inhibition of harmful bacteria [22]. In the present study, beneficial bacteria were detected in the stomach samples of both native and exotic pigs, whereas most harmful bacteria were not detected. This could be due to the lower pH value in the stomach of pigs [23].

Both beneficial bacteria (*Bifidobacteria* and *Lactobacillus*) and harmful bacteria (*Clostridium* spp.) were detected in the ileum of native and exotic pigs. This result suggested that microbiota compositions in the ileum have changed compared with those in the stomach. A recent study by Konstantinov *et al*. [24] reported that the neutral pH in the ileum was a good condition for the development of harmful bacteria. Among the native pigs fed with the HFC diet, three pigs showed the presence of the DNA band h64 with matching similarity with uncultured bacteria. This may be because the HFC diet with highly soluble NSP contained positive substrates for those bacterial species. Moreover, the DNA bands h105 and h99 appeared in the ileum samples of exotic pigs fed with HFC and HFB diets, suggesting that an increase in fiber level in the diet led to the development of *Lactococcus garvieae* and *L. lactis*. A previous study [25] reported that *L*. *garvieae* has been increasingly considered as an emerging zoonotic pathogen. In the ileum of exotic pigs, an increase in the abundance of harmful bacteria such as *Streptococcus*, *L. garvieae*, and *Clostridium* and a decrease in that of beneficial bacteria such as *V. magna*, *Bifidobacterium*, and *L. lactis* were detected as DNA bands, consistent with a previous study [23] that indicated that the abundance of beneficial bacteria in the ileum of pigs was lower than that of harmful bacteria.

The DNA bands k141 and k201 detected in the colon of both native and exotic pigs fed with different diets matched with the sequences of the beneficial bacteria *Lactobacillus helveticus*, *L. gallinarum*, *L*. *crispatus*, and *L. amylovorus*, whereas the remaining DNA bands were not clear and difficult for observation. Consequently, *Lactobacillus* spp. were common bacteria in the colon of pigs. Furthermore, in the colon of native pigs fed with the HFB diet, the DNA band k131 showed 98% similarity with the sequence of *Butyrivibrio fibrisolvens*, and the exotic pigs fed with the experimental diets showed the DNA band k175 with 98% similarity with the sequence of *B. fibrisolvens*. In the colon of native pigs fed with the experimental diets, DNA bands matching with *Ruminococcus* spp. were detected, but they were not detected in exotic pigs. A previous study by Varel *et al*. [26] reported that pigs fed with a high-fiber diet showed an increased abundance of beneficial bacteria that could digest cellulose and hemicellulose, such as *Bacteroides succinogenes*, *B. intestinalis*, *Ruminococcus flavefaciens*, *R. albus*, *Butyrivibrio*, and *P. ruminicola*. Increasing the fiber content in the diet to up to 35% led to an increased number of *Ruminococcus* and *Bacteroides* [27]. Carbohydrates are the primary energy source for fermentation in the large intestine [28], and therefore, the types of fiber in pig diets influence the density and composition of gut microbiota [18,29-31]. Moreover, the microbiota in the digestive tract of pigs is modulated by increasing a bacterial community that could digest NSP ­fractions [32]. Both beneficial bacteria and harmful bacteria were also detected in the colon of native and exotic pigs. The number of sequences of DNA bands in the colon of pigs that matched with uncultured bacteria was higher than that of sequences that matched with uncultured bacteria in the ileum.

## Conclusion

This study indicated that the number of DNA bands in the stomach samples and the Dice coefficient of gene diversity were higher in exotic pigs than in native pigs. The high-fiber diet resulted in a higher number of DNA bands in the ileum of exotic pigs than that in native pigs. High-fiber levels in the diet enhanced the number of beneficial bacteria in the colon of both pig breeds, and beneficial bacteria (*Bifidobacterium* in the stomach and *L. lactis* in the ileum) were detected in the exotic pigs, whereas *Ruminococcus* spp. was detected in the native pigs.

## Data Availability

Supplementary data can be available from the corresponding author on a reasonable request.

## Authors’ Contributions

TTBN, TTTH, PKD, and NCO: Conceived and designed the study. TTBN and TTTH: Conducted the experiment and collected the data. TTBN and TTTH: Analyzed the samples. NCO and TTBN: Analyzed the data. TTBN and NCO: Drafted the manuscript. All authors read, revised, and approved the manuscript.
